# Exercise heat tolerance assessment following a diagnosis of heat illness in UK military personnel

**DOI:** 10.1186/2046-7648-4-S1-A105

**Published:** 2015-09-14

**Authors:** Dan Roiz de Sa, Carol House

**Affiliations:** 1 Environmental Medicine and Science, Institute of Naval Medicine, Gosport, UK

## Introduction

Exertional heat stroke and heat illness (HI) continue to pose a significant threat to military training and operations in both temperate and hot climates. In 2001, the Institute of Naval Medicine (INM) established a formal protocol for Heat Tolerance Testing (HTT) and in 2003 it became UK Armed Forces Policy that personnel who have been diagnosed as having suffered from an episode of heat stroke or HI with a significant biochemical disturbance or multiple episodes of HI are referred to the Heat Illness Clinic (HIC) at the INM. Following a full medical examination and assessment of maximal aerobic fitness (VO_2max_) patients are asked to exercise on a treadmill at 60% VO_2max _at 34°C dry bulb, relative humidity 40% (WBGT of 27 °C) with clothing limitations and load carriage to initially raise the deep body temperature of the individual. At 30 minutes the jacket and weighted rucksack are removed and at 45 minutes the remaining t-shirt is also removed. Patients continue to exercise for a minimum of 60 minutes or up to 90 minutes duration to determine whether thermal equilibrium (*i.e*. a plateau of rectal temperature) can be achieved. Patients achieving thermal balance are considered to show normal thermoregulation (and pass HTT), those who do not or have another medical issue identified are considered to be heat intolerant (fail). Those who do not pass the assessment are reviewed at least once a minimum of 8 weeks later. Measurements of rectal temperature, skin temperature, heart rate and sweat rate made during the HHT are used to determine whether an individual's heat intolerance relates to an abnormal level of heat production (abnormal muscle) or impaired heat dissipation mechanisms.

## Methods

Patient outcomes of the HTT reported for those who attended in 2014 were reviewed.

## Results

In 2014, 140 patients were assessed in the INM HIC, assessment outcomes are shown in Table 1. Of the patients that failed their first HTT, one was diagnosed with hyperventilation problems, one recommended for psychological support and the remaining five required reassessment. Four patients failed on two or more occasions and were recommended further investigation for malignant hyperthermia (MH) sensitivity.

**Table 1:** Outcome of the HTT in the 140 visiting the HIC in 2014.

	Males	Females	Total
Passed first HTT (Recommended Return To Military Activity)	116	6	122

Passed second HTT (first HTT was in 2013)	2	1	3

Failed first HTT (Remain Downgraded)	7	0	7

Failed HTT twice (insufficient aerobic fitness)	1	1	2

Failed first and passed retest	2	0	2

Failed and recommended for MH testing	4	0	4

## Conclusion

Only a small number of the HI casualties referred to the HIC demonstrate abnormal thermoregulation. Although the HTT appears to discriminate between individuals with normal and abnormal thermoregulation further work is required to confirm the specificity and sensitivity of the HTT.

